# Analysis and comparison of the trends in burden of rheumatic heart disease in China and worldwide from 1990 to 2019

**DOI:** 10.1186/s12872-023-03552-w

**Published:** 2023-10-24

**Authors:** Lang Shi, Chenglu Bao, Ya Wen, Xuehui Liu, Guiying You

**Affiliations:** Department of Cardiology, West China Hospital, Sichuan University/West China School of Nursing, Sichuan University, NO.37, Lane outside the southern, Chengdu, 610000 Sichuan China

**Keywords:** Rheumatic Heart Disease, Trend, Incidence, Mortality, Prevalence, Disability-adjusted life years

## Abstract

**Objectives:**

This study aimed to describe the temporal trends in age and gender burdens of rheumatic heart disease (RHD) in China from 1990 to 2019, including incidence, prevalence, mortality, and disability-adjusted life years (DALYs), and to compare them with the global burden of the disease.

**Methods:**

Using open data from the Global Burden of Disease (GBD) database from 1990 to 2019, this study analyzed the characteristics of RHD burden in China and worldwide, including changes in incidence, prevalence, mortality, and DALYs. Joinpoint was used to calculate the average annual percentage change (AAPC) and the corresponding 95% confidence interval (95% CI) to reflect the trends in the burden of RHD. A comprehensive comparative analysis of the differences in RHD burden between China and the rest of the world was conducted from multiple dimensions, including age, gender, and time periods.

**Results:**

From 1990 to 2019, the age-standardized incidence rate (ASIR) of RHD in China decreased from 29.62/100,000 to 23.95/100,000, while the global ASIR increased from 32.69/100,000 to 37.40/100,000. The age-standardized prevalence rate (ASPR) in China decreased from 446.15/100,000 to 390.24/100,000, while the global ASPR increased from 451.56/100,000 to 513.68/100,000. The age-standardized rates of mortality (ASMR) in China decreased from 18.11/100,000 to 4.04/100,000, while the global ASMR decreased from 8.94/100,000 to 3.85/100,000. The age-standardized DALY rate (ASDR) in China decreased from 431.45/100,000 to 93.73/100,000, while the global ASDR decreased from 283.30/100,000 to 132.88/100,000. The AAPC of ASIR, ASPR, ASMR, and ASDR in China was − 0.73%, -0.47%, -5.10%, and − 5.21%, respectively, while the AAPC of the global burden of RHD was 0.48%, 0.45%, -2.87%, and − 2.58%, respectively. The effects of age and gender on the burden of RHD were different. ASIR generally decreased with increasing age, while ASPR increased first and then decreased. ASMR and ASDR increased with increasing age. Women had higher incidence and mortality rates of RHD than men.

**Conclusion:**

From 1990 to 2019, the incidence, prevalence, mortality, and DALYs of RHD in China decreased, indicating a relative reduction in the burden of RHD in China. The burden of RHD is age-related, with a higher prevalence observed in the younger population, a peak incidence among young adults, and a higher mortality rate among the elderly population. Women are more susceptible to RHD and have a higher risk of mortality than men. Given China’s large population and aging population, RHD remains a significant public health challenge in China.

**Supplementary Information:**

The online version contains supplementary material available at 10.1186/s12872-023-03552-w.

## Introduction

Rheumatic heart disease (RHD) is an acquired heart disease commonly seen in children and adolescents caused by group A streptococcal infections [1]. According to the latest results of the Global Burden of Disease (GBD), RHD is estimated to affect 400,000 people, and the global number of RHD deaths in 2019 was approximately 310,000 [1]. Studies have found that the burden of RHD is influenced by multiple related factors, mainly genetic, environmental, and socioeconomic factors [2].

Although RHD is considered preventable, it remains an important public health problem worldwide due to its early onset, high mortality rate, and disability rate, especially in many low-income countries [3]. Over the past two decades, significant progress has been made in controlling RHD worldwide, with a 50% decrease in RHD mortality rate, but the rate of decline varies greatly among countries and regions [4]. For example, from 1990 to 2015, the age-standardized RHD mortality rate in India decreased by 18%, while in China during the same period, it decreased from 18.8 to 5.4 per 100,000 population, a decrease of 71% [5]. This reflects the close relationship between RHD mortality rate and environmental and socio-economic conditions. To reduce the mortality rate of RHD, countries affected by RHD need to systematically implement evidence-based prevention and treatment strategies, rigorously evaluate potential strategies and their cost-effectiveness to assist in further reducing mortality rates. In addition, advances in diagnostic and surgical techniques have also promoted changes in the epidemiological pattern of RHD [1]. Therefore, tracking the temporal trends of RHD burden has become a necessary condition for health strategies.

Current reports on the burden of RHD from GBD studies have mainly focused on macro-level assessments at global and regional levels. Existing research has estimated global trends and attributable risks of RHD from 1990 to 2019 [1, 6], examined their relationship with socio-economic development status [2], and provided future predictions on RHD burden [7]. However, these studies have primarily approached the issue from a global perspective, failing to delve deeper into the heterogeneity among different countries and regions, thus missing the specific circumstances of specific countries. China, as the world’s most populous country, has garnered considerable attention in the medical community due to the burden imposed by RHD. While there have been some related studies on the burden of RHD in China [8], these analyses have mainly focused on trends in deaths and Disability-adjusted Life Years (DALYs) attributed to RHD. A more comprehensive investigation into the progression of RHD within the Chinese population has not been thoroughly conducted. Therefore, this study, based on the latest GBD data, comprehensively analyzes and compared the burden of RHD in China and worldwide from 1990 to 2019. We employed Joinpoint regression analysis to explore the temporal trends in RHD and conducted an in-depth examination of the burden changes over three decades from both age and gender perspectives. The aim is to provide valuable insights for decision-makers in assessing the overall burden of RHD in China and to facilitate the development of targeted prevention strategies and the equitable allocation of public health resources.

## Methods

### Data source

The data used in this study were extracted from the GBD 2019 dataset, which is a comprehensive database recording the incidence, prevalence, and mortality rates of over 300 diseases and injuries across 204 countries and regions, categorized by age and gender. The GBD dataset is compiled from various sources, including 46,749 cohort studies, randomized controlled trials, civil surveys, and other research studies [9]. It summarizes and quantifies risks and exposures. RHD is a cardiac condition primarily affecting heart valves, particularly the mitral valve. It stems from acute rheumatic fever triggered by Group A Streptococcus infection and is known to often result in premature mortality and heart failure [GBD cause code B.2.1, ICD-10 codes: 101-101.9, 102.0, 105-109.9] [7].

For most diseases and injuries, modeling of processed data is carried out using standardized tools to estimate study subjects by age, gender, location, and year. In this study, DisMod-MR and the Cause of Death Ensemble model (CODEm) served as the primary standardization tools. DisMod-MR is a Bayesian meta-regression tool that assesses all available data on the incidence, prevalence, remission, and mortality rates of one disease while enhancing consistency among epidemiological parameters [10]. CODEm is a highly systematic tool employed for analyzing cause of death data. It employs a collection of different modeling methods to analyze ratios or cause fractions and selects various covariates that perform optimally in out-of-sample predictive validity testing [11, 12].

The data related to RHD in this study were sourced from the Global Health Data Exchange (GHDx) and its affiliated tools (http://ghdx.healthdata.org/gbd-results-tool). We utilized the GBD tool to extract data on the incidence rate, prevalence rate, mortality rate, and DALYs for China and the world from 1990 to 2019, which served as the metrics for assessing the burden of RHD. The institutional ethics committee granted an exemption for this study, as it did not require approval, given that the data from the 2019 GBD are publicly available. This study adhered to the guidelines for accurate and transparent health assessment reporting.

### Statistical analysis

We screened the incidence, prevalence, and mortality, DALYs, and corresponding age-standardized incidence rate (ASIR), age-standardized prevalence rate (ASPR), age-standardized rates of mortality (ASMR), and age-standardized DALY rate (ASDR) of RHD in China and worldwide from the GBD database, as well as the crude incidence rate (CIR), crude prevalence rate (CPR), crude mortality rate (CMR), and crude DALY rate (CDR) for each age group. The average annual percentage change (AAPC) and corresponding 95% confidence interval (95% CI) were calculated using Joinpoint software (National Cancer Institute, Rockville, MD, USA) to determine the burden trend of the disease [13, 14]. The logarithmic age-standardized indicators can be fitted into a regression model, i.e., ln(y)=α + βx + ε, where y represents the respective age-standardized indicator and x represents the calendar year. The AAPC was calculated as 100 × (exp(β) − 1), and the 95% CI can also be calculated from the model. If the 95% CI of the corresponding AAPC estimate is > 0, the age-standardized indicator shows an increasing trend; if < 0, it shows a decreasing trend; if it includes 0, it shows a stable trend.

The statistical analysis and visualization of the data in this study were performed using R statistical software program (version 4.1.3) and Joinpoint software program (version 4.9.1.0). A *P* value < 0.05 was considered to be statistically significant.

## Results

### Description of the burden of RHD in China and worldwide

#### Incidence of RHD in China and worldwide

The number of RHD cases in China decreased from 369,383 (95% CI: 282,677 − 460,322) in 1990 to 267,676 (95% CI: 215,457 − 327,586) in 2019, representing a cumulative reduction of 27.53%. However, globally, the incidence increased from 1,863,318 cases (95% CI: 1,438,465-2,308,707) in 1990 to 2,789,443 cases (95% CI: 2,153,319-3,454,256) in 2019, representing a cumulative increase of 49.70%. The ASIR globally increased from 32.69 (95% CI: 25.77–40.03) per 100,000 population in 1990 to 37.40 (95% CI: 28.60-46.74) per 100,000 population in 2019. In China, the ASIR decreased from 29.62 (95% CI: 23.08–36.86) per 100,000 population in 1990 to 23.95 (95% CI: 18.62–29.89) per 100,000 population in 2019. Meanwhile, the AAPC of the incidence rate in China decreased by 0.73% (95% CI: -0.76 to -0.71) from 1990 to 2019, while it increased by 0.48% (95% CI: 0.45–0.50) globally (Table 1).

**Table 1:** All-age cases and age-standardized incidence, prevalence, mortality, and DALYs rates and corresponding AAPC of RHD in China and globally in 1990 and 2019

Location	Measure	1990		2019		1990–2019 AAPC
		All-ages cases	Age-standardized rates per 100,000 people	All-ages cases	Age-standardized rates per 100,000 people	
		n (95% CI)	n (95% CI)	n (95% CI)	n (95% CI)	n (95% CI)
China	Incidence	369,383 (282,677–460,322)	29.62 (23.08–36.86)	267,676 (215,457–327,586)	23.95 (18.62–29.89)	-0.73 (-0.76 - -0.71)
	Prevalence	5,557,179 (4,292,771–6,980,851)	446.15 (352.68-556.93)	5,981,816 (4,858,192–7,347,719)	390.24 (310.29-481.99)	-0.47 (-0.52 - -0.41)
	Deaths	132,665 (114,115–155,349)	18.11 (15.52–21.27)	69,782 (57,365–81,361)	4.04 (3.32–4.69)	-5.10 (-5.44 - -4.77)
	DALYs	4,019,191 (3,460,824–4,669,340)	431.44 (373.65-499.87)	1,705,942 (1,424,796–1,984,609)	93.73 (78.43-108.78)	-5.21 (-5.44 - -4.97)
Global	Incidence	1,863,318 (1,438,465–2,308,707)	32.69 (25.77–40.03)	2,789,443 (2,153,319–3,454,256)	37.40 (28.6-46.74)	0.48 (0.45–0.50)
	Prevalence	23,756,847 (18,791,683–29,295,709)	451.56 (363.35-552.54)	40,502,345 (32,052,904–50,062,426)	513.68 (405.01-636.25)	0.45 (0.42–0.48)
	Deaths	362,160 (326,259–408,222)	8.94 (8.04–10.12)	305,651 (259,220–340,486)	3.85 (3.29–4.29)	-2.87 (-2.99 - -2.76)
	DALYs	13,168,339 (11,896,460–14,634,663)	283.30 (255.92-315.25)	10,673,882 (9,207,379–12,121,608)	132.88 (115.02-150.34)	-2.58 (-2.69 - -2.46)

#### Prevalence of RHD in China and worldwide

In terms of prevalence, the number of RHD cases in China increased from 5,557,179 (95% CI: 4,292,771-6,980,851) in 1990 to 5,981,816 (95% CI: 4,858,192-7,347,719) in 2019, representing a cumulative increase of 7.64%. However, globally, the prevalence increased from 23,756,847 (95% CI: 18,791,683 − 29,295,709) in 1990 to 40,502,345 (95% CI: 32,052,904 − 50,062,426) in 2019, representing a cumulative increase of 70.49%. The ASPR globally increased from 451.56 (95% CI: 363.35-552.54) per 100,000 population in 1990 to 513.68 (95% CI: 405.01-636.25) per 100,000 population in 2019. In China, the ASPR decreased from 446.15 (95% CI: 352.68-556.93) per 100,000 population in 1990 to 390.244 (95% CI: 310.29-481.99) per 100,000 population in 2019. Meanwhile, the AAPC of the prevalence globally increased by 0.45% (95% CI: 0.42–0.48) from 1990 to 2019, while it decreased by 0.47% (95% CI: -0.52 to -0.41) in China (Table 1).

#### Mortality of RHD in China and worldwide

Globally, RHD caused 305,651 (95% CI: 259,220–340,486) deaths in 2019, representing a 15.60% decrease compared to 1990. In China, the mortality rate decreased by 47.40% from 1990 to 2019. The ASMR globally decreased from 8.94 (95% CI: 8.04–10.12) per 100,000 population in 1990 to 3.85 (95% CI: 3.29–4.29) per 100,000 population in 2019. In China, the ASMR decreased from 18.11 (95% CI: 15.52–21.27) per 100,000 population in 1990 to 4.04 (95% CI: 3.32–4.69) per 100,000 population in 2019. Meanwhile, the AAPC of the mortality rate globally decreased by 2.87% (95% CI: -2.99 to -2.76) from 1990 to 2019, while it decreased by 5.10% (95% CI: -5.44 to -4.77) in China (Table 1).

#### DALYs of RHD in China and worldwide

Globally, the DALYs for RHD were 13,168,339 (95% CI: 11,896,460 − 14,634,663) in 1990 and 10,673,882 (95% CI: 9,207,379 − 12,121,608) in 2019, representing an 18.94% decrease compared to 2019. In China, the DALYs decreased by 57.56% from 1990 to 2019. The ASDR globally decreased from 283.30 (95% CI: 255.92-315.25) per 100,000 population in 1990 to 132.88 (95% CI: 115.02-150.34) per 100,000 population in 2019. In China, the ASDR decreased from 431.45 (95% CI: 373.65-499.87) per 100,000 population in 1990 to 93.73 (95% CI: 78.43-108.78) per 100,000 population in 2019. Meanwhile, the AAPC of the DALYs globally decreased by 2.58% (95% CI: -2.69 to -2.46) from 1990 to 2019, while it decreased by 5.21% (95% CI: -5.44 to -4.97) in China (Table 1).

### Joinpoint regression analysis of the burden of RHD in China and worldwide

**Fig. 1:**
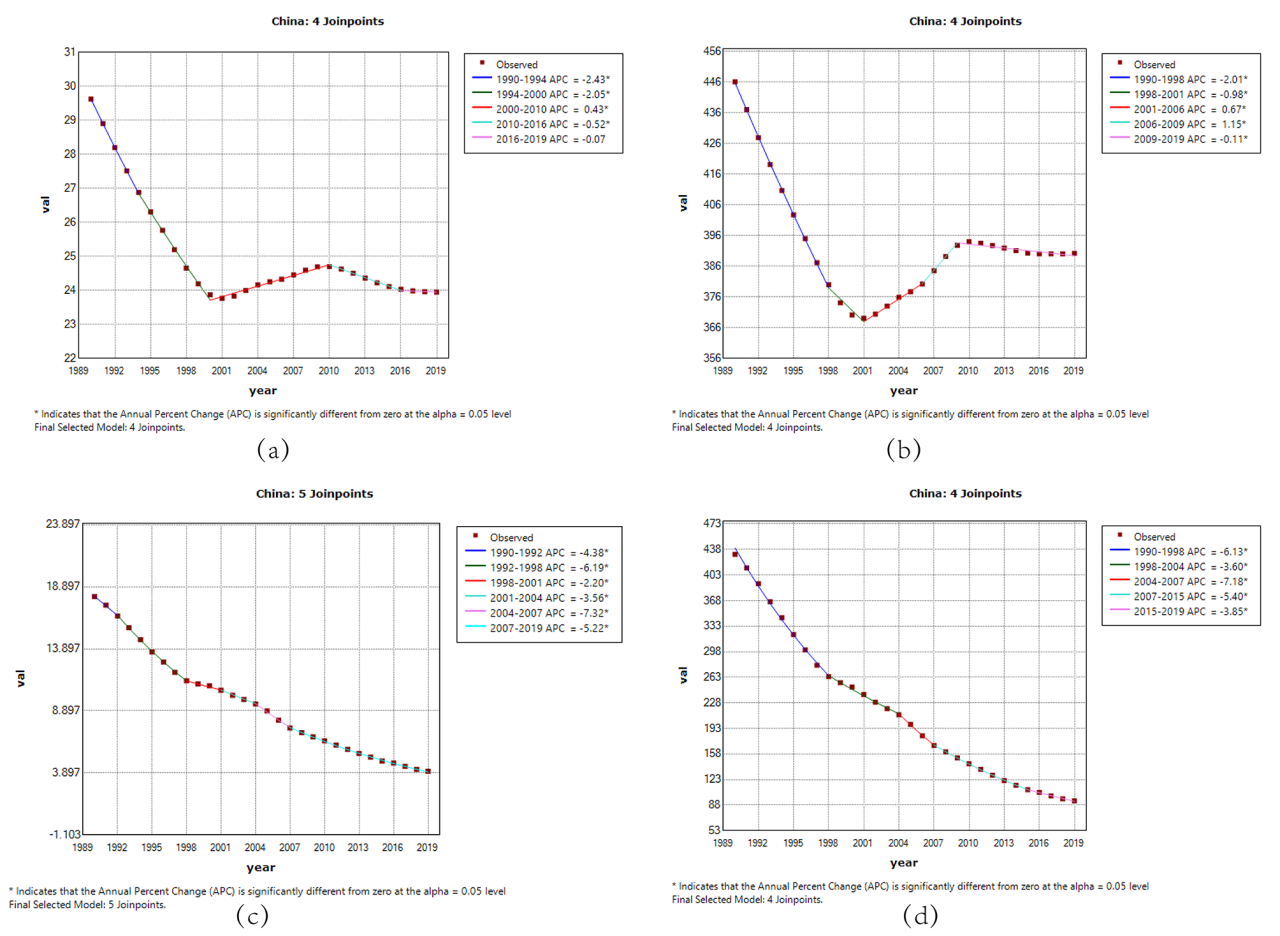
The APC of ASIR, ASPR, ASMR, and ASDR of RHD in China from 1990 to 2019 (* means p-values < 0.05 and significant results). (**a**) ASIR; (**b**) ASPR; (**c**) ASMR; (**d**) ASDR

**Fig. 2:**
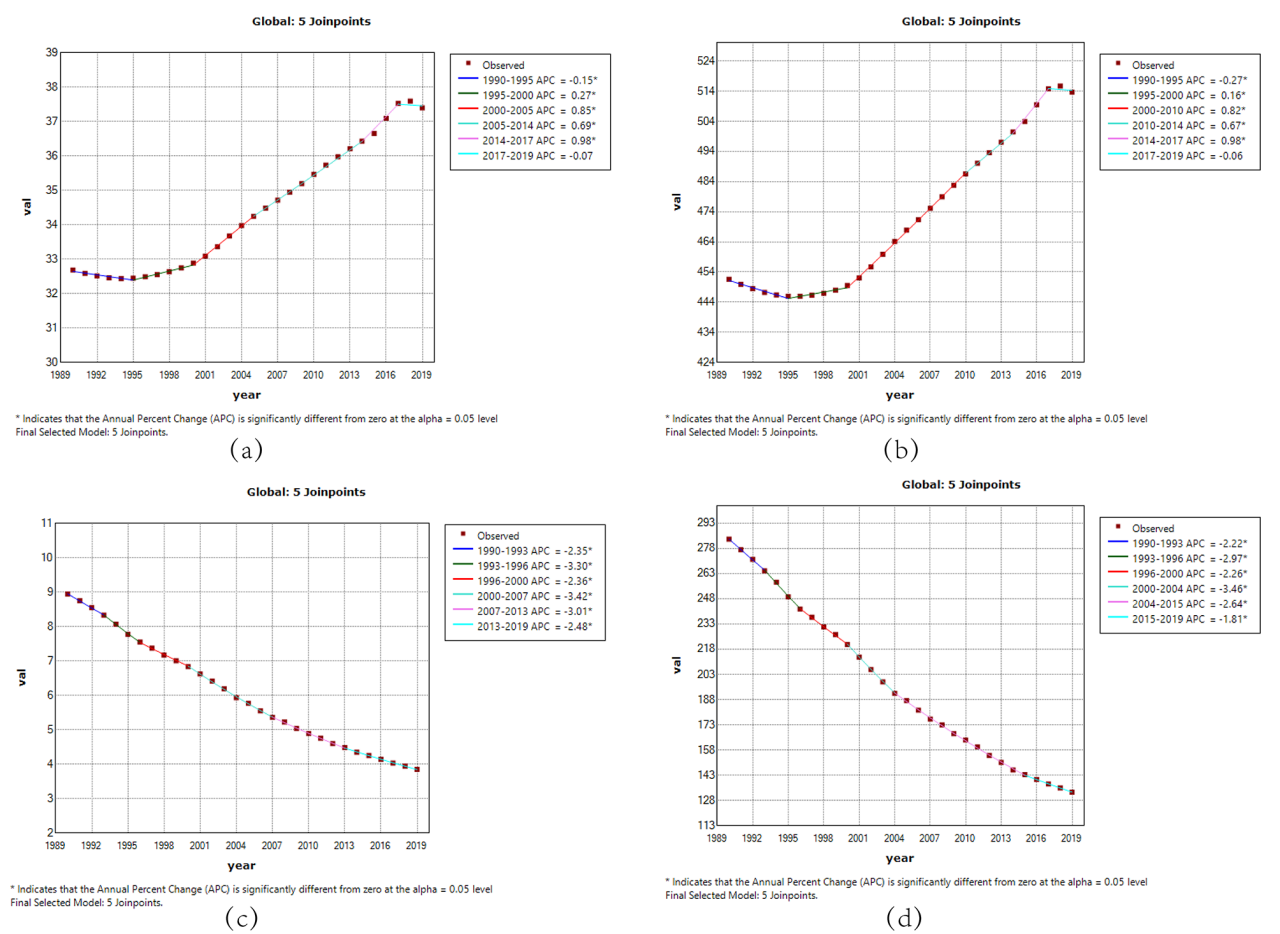
The APC of ASIR, ASPR, ASMR, and ASDR of RHD in Global from 1990 to 2019 (* means p-values < 0.05 and significant results). (**a**) ASIR; (**b**) ASPR; (**c**) ASMR; (**d**) ASDR

The Joinpoint regression analysis of ASIR, ASPR, ASMR, and DALYs for RHD in China and worldwide from 1990 to 2019 is depicted in Figs. 1 and 2. The annual percentage change (APC) for RHD ASIR and ASPR in China showed significant declines during the period 1990 to 2000 (ASIR: 1990–1994 APC = -2.43; 1994–2000 APC = -2.05, *P* < 0.05; ASPR: 1990–1998 APC = -2.01; 1998–2001 APC = -0.98, *P* < 0.05). However, there was an upward trend from 2000 to 2010, followed by a slight decrease after 2010, showing fluctuations. Globally, ASIR and ASPR exhibited an overall significant increase after 1995 (*P* < 0.05), with a slight decrease observed in 2017–2019. Over the period 1990 to 2019, both China and global ASMR for RHD showed significant declines (*P* < 0.05).

### Trends in the burden of RHD disease in China and worldwide

**Fig. 3:**
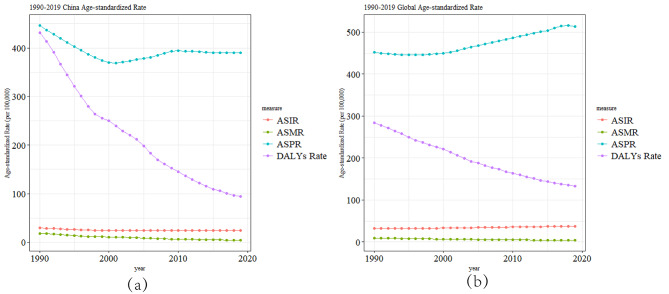
Trend comparison of ASIR, ASPR, ASMR, and ASDR of RHD in China and worldwide from 1990 to 2019

The ASDR of RHD in China and worldwide has gradually decreased from 1990 to 2019, with a significantly greater decline in China. Meanwhile, the trend of the ASPR of RHD in China showed a significant decrease from 1990 to 2000, followed by a slow increase after 2000 and a flattening trend after 2010, showing an overall decreasing trend. In contrast, the ASPR of RHD globally showed a flat trend from 1990 to 2000 and an increase after 2000. In addition, the ASIR and ASMR of RHD in China showed a slight decreasing trend from 1990 to 2019, while the ASIR of RHD globally showed a slight increasing trend, and the ASMR showed a slight decreasing trend (Fig. 3).

### Burden of RHD in different age groups in China in 1990 and 2019

**Fig. 4:**
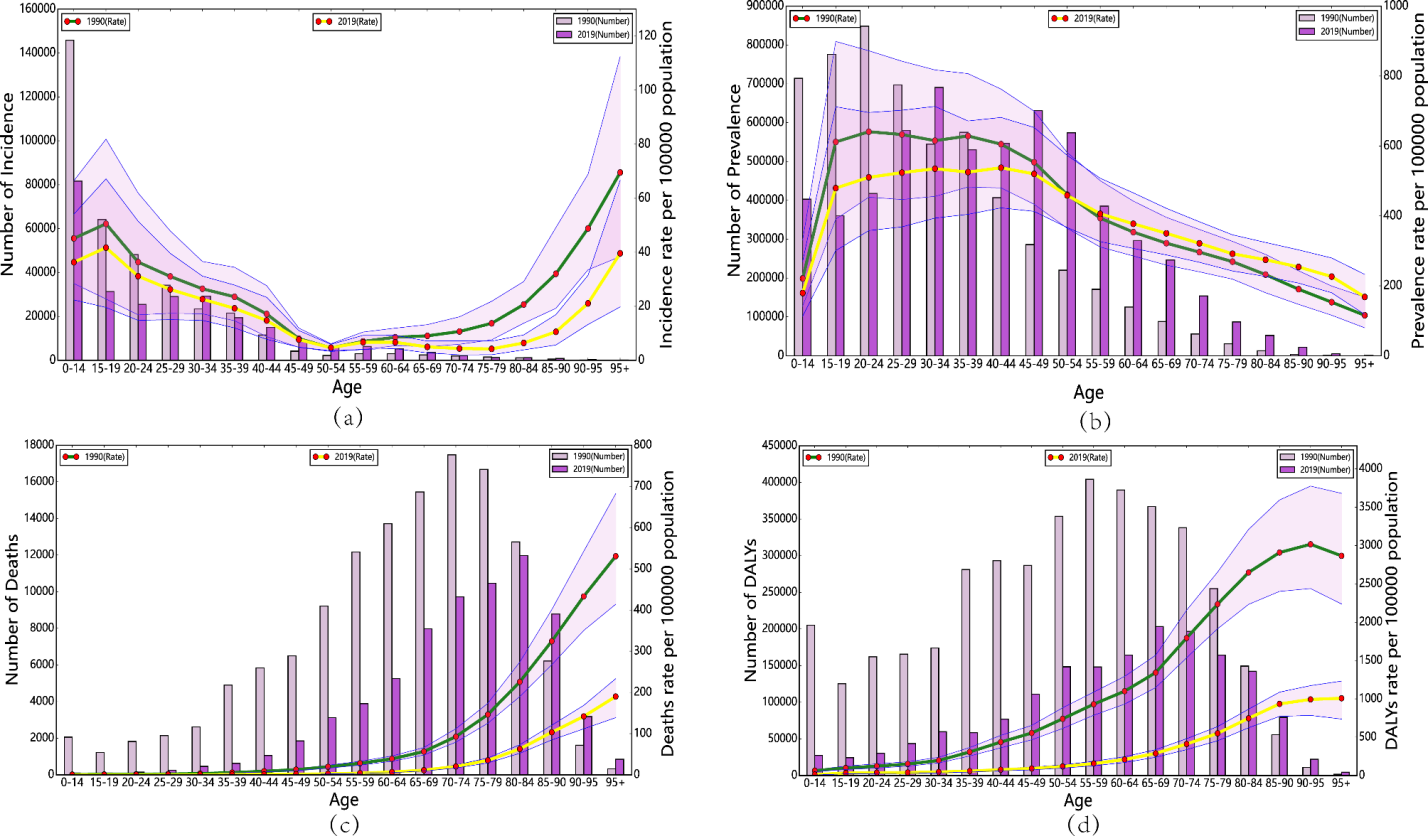
Comparative of the incidence, prevalence, deaths, and DALYs counts, along with their crude rates, by age group in China from 1990 and 2019. (**a**) Incident cases and CIR; (**b**) Prevalent cases and CPR; (**c**) Death cases and CMR; (**d**) DALYs counts and CDR; Bar charts represent counts; lines represent crude rates

Figure 4 exhibited a comparison of the incidence, prevalence, mortality, and DALYs of RHD in different age groups in China in 1990 and 2019, along with their corresponding crude rates. From the incidence rate results, RHD was prevalent among people under 50 years old, with the highest number of cases in the 0–35 age group. Both in 1990 and 2019, the CIR of RHD in China showed an increasing trend from the 0–14 age group to the 15–19 age group, and a decreasing trend between the ages of 20–54, with the highest incidence peak occurring between the ages of 0–14. In contrast, the global CIR of RHD showed an increasing trend in all age groups, with a higher incidence rate than in China (Fig. 4a, Supplementary Fig. 1). Similar trends were observed in CPR (Supplementary Fig. 1). In 1990, the peak prevalence of RHD in China occurred in the 20–24 age group, while in 2019, it occurred in the 30–34 age group (Fig. 4b). Regarding deaths, the age group with the highest number of deaths was 70–74 in 1990, and 80–84 in 2019. The CMR of RHD increased with age and showed an increasing trend. The age group with the highest mortality rate was 95 + in both 1990 and 2019 (Fig. 4c). Similar trends were observed in CDR, which increased with age. In 1990, the peak DALYs occurred in the 55–59 age group, while in 2019, it occurred in the 65–69 age group (Fig. 4d), which was similar to the changing trends in global CDR and CMR (Supplementary Fig. 1).

### Gender disparities in the burden of RHD in different age groups in China

**Fig. 5:**
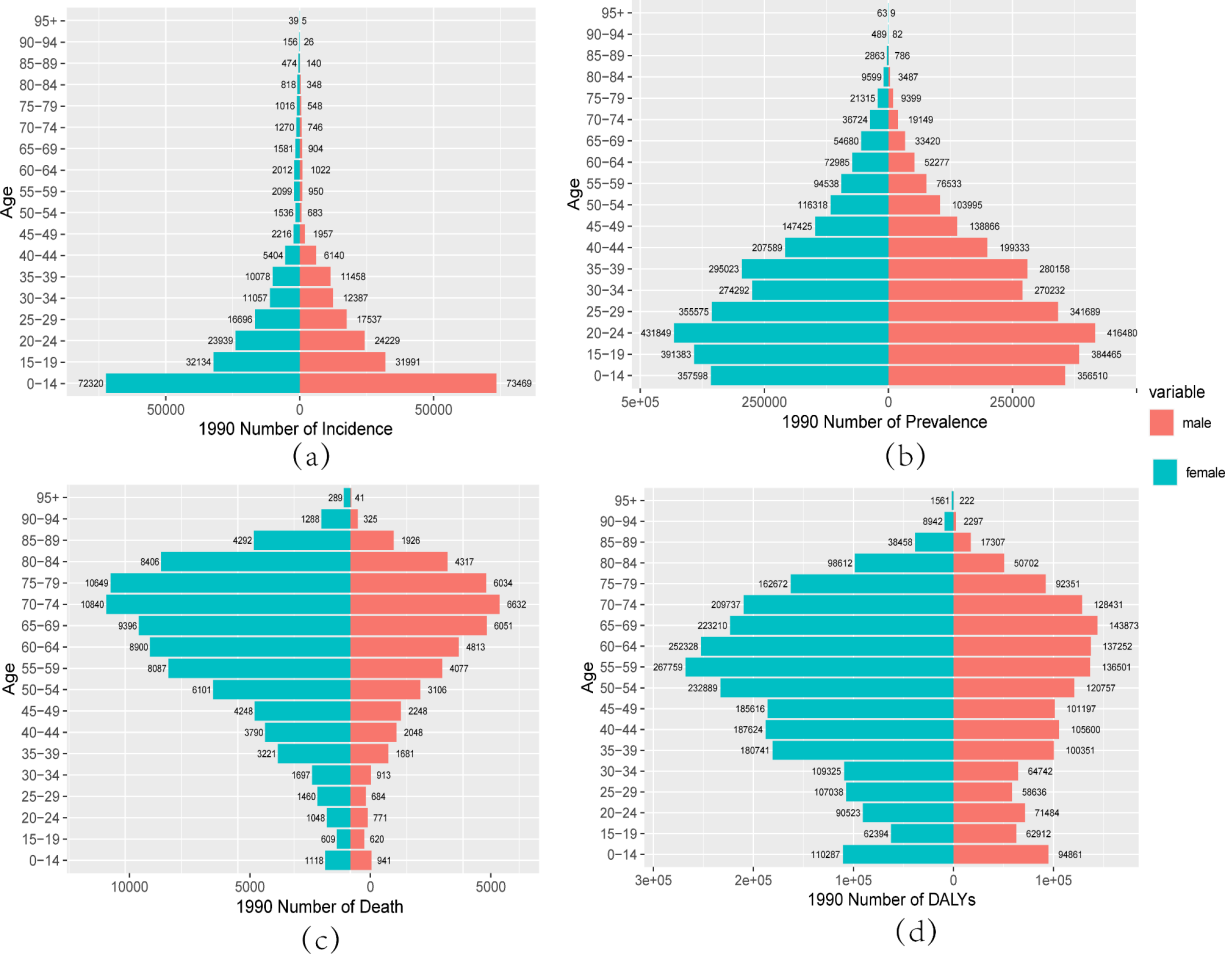
Comparison of the number of incidence, prevalence, mortality, and DALYs of RHD in males and females of different age groups in China in 1990. (**a**) Incidence; (**b**) Prevalence; (**c**) Mortality; (**d**) DALYs

**Fig. 6:**
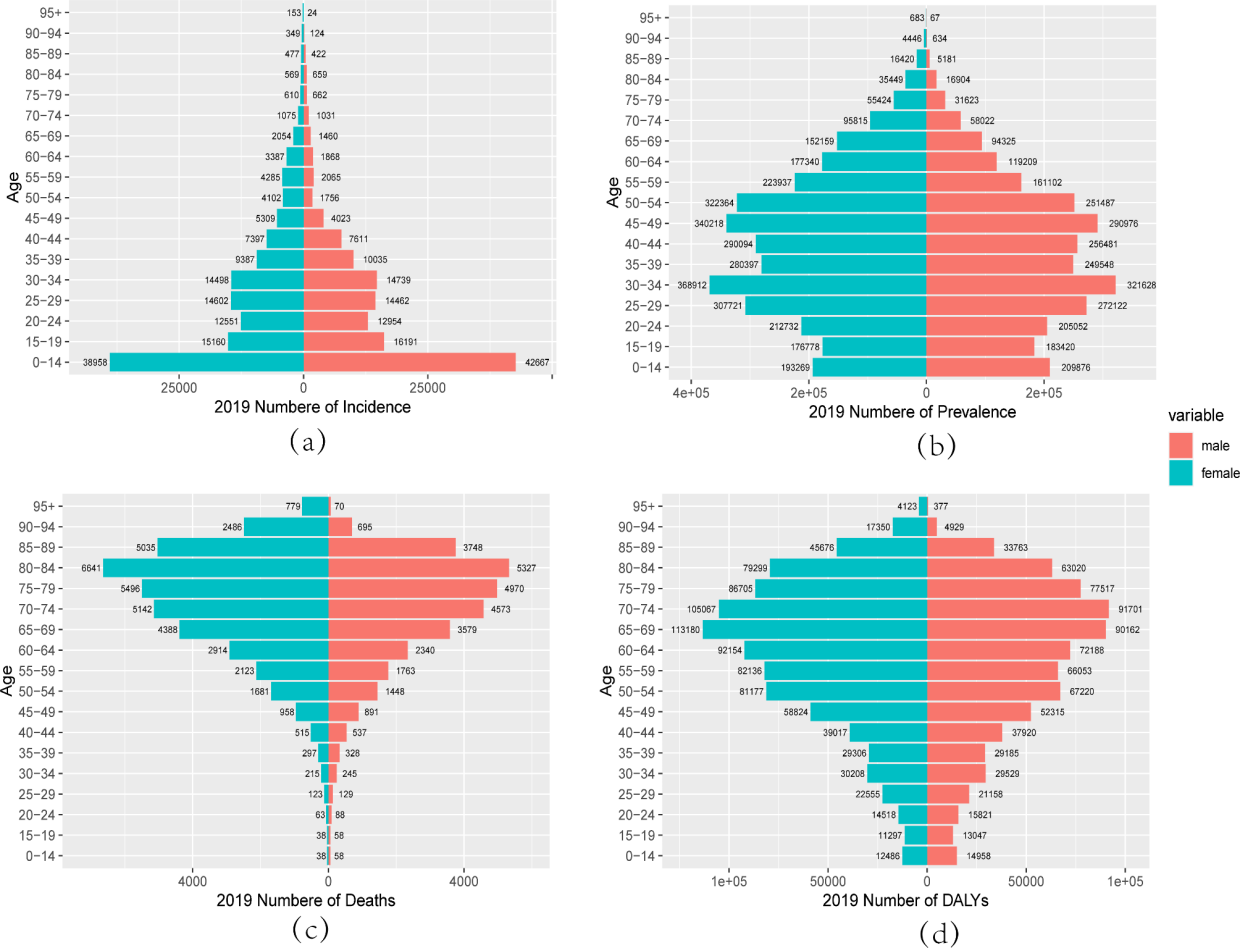
Comparison of of the number incidence, prevalence, mortality, and DALYs of RHD in males and females of different age groups in China in 2019. (**a**) Incidence; (**b**) Prevalence; (**c**) Mortality; (**d**) DALYs

Figures 5 and 6 illustrate the incidence, prevalence, mortality, and DALYs of RHD in different age groups of males and females in China in 1990 and 2019. From the incidence results, the peak incidence of RHD in both males and females occurred in the 0–14 age group, and the incidence rate decreased with age after 14. In 1990, except for the 15–19 age group, the number of males with RHD was higher than that of females in all age groups below 45, while females had a higher number of cases after 45. In contrast, in 2019, the number of males with RHD was higher than that of females in all age groups below 50, and females had a higher number of cases after 50 (Figs. 5a and 6a). From the prevalence results in 1990, the number of males and females with RHD increased from the 0–14 age group to the 20–24 age group, with the highest prevalence peak occurring in the 20–24 age group. The number of cases decreased with age after 25, which was opposite to the global trend, where the number of cases increased with age (Supplementary Fig. 2). In 1990, females had a higher number of cases than males in all age groups, while in 2019, except for the 0–19 age group, females had a higher number of cases than males in all other age groups, reaching a peak in the 30–34 age group (Figs. 5b and 6b). Moreover, when comparing the number of deaths between males and females in 1990, females had a higher number of deaths than males in all age groups, and the number of deaths for both genders peaked at 70–74 years old. In 2019, the number of deaths for females was higher than that for males in the elderly population over 50, and the peak period for deaths was 80–84 years old. The mortality of RHD increased with age for both genders, reaching a peak and then gradually decreasing, while the global number of deaths increased with age (Figs. 5c and 6c, Supplementary Fig. 2). The results of DALYs were similar to those of mortality, with females having a higher number of DALYs than males in all age groups. In 1990, the peak DALYs for females occurred in the 55–59 age group, while for males, it occurred in the 65–69 age group. In 2019, the peak DALYs for females occurred in the 65–69 age group, while for males, it occurred in the 70–74 age group (Figs. 5d and 6d).

**Fig. 7:**
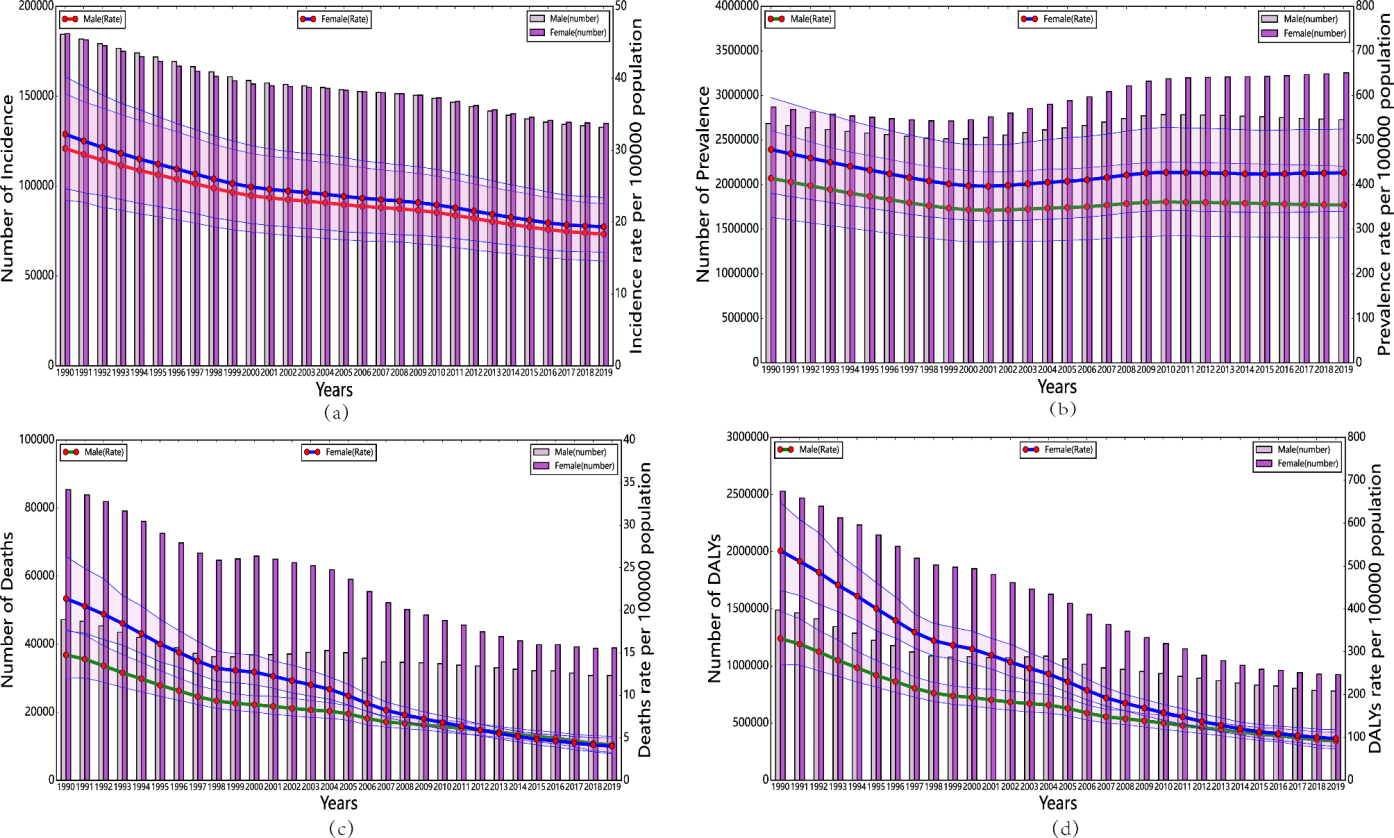
Comparison of full-age cases and age-standardized rates of incidence, prevalence, mortality and DALYs among men and women in China from 1990 to 2019. (**a**) Incident cases and ASIR; (**b**) Prevalent cases and ASPR; (**c**) Death cases and ASMR; (**d**) DALYs counts and ASDR. Bar charts represent counts; lines represent age-standardized rates

Figure 7 displayed a comparison of the disease burden and age-standardized rates of RHD in males and females of all ages in China from 1990 to 2019. Figure 7a shows the ASIR of RHD in males and females, which reached its peak in 1990 with the largest difference between the two genders. The ASIR then decreased with increasing years, and the difference between males and females also decreased. The ASPR of RHD in males and females gradually decreased from 1990 to 2001, slightly increased from 2002 to 2009, and then leveled off after 2010. The ASPR in females was consistently higher than that in males (Fig. 7b). In contrast, the ASIR and ASPR of RHD in males and females globally increased with increasing years, which was opposite to the trend in China (Supplementary Fig. 3). Moreover, Fig. 7c shows that the number of deaths and ASMR of RHD in males and females of all ages differed significantly in 1990, with females having a significantly higher ASMR than males. The difference between the two genders gradually decreased over time, and the overall ASMR decreased. The ASDR of RHD had similar results to those of ASMR, and the trend was consistent with that globally (Fig. 7d, Supplementary Fig. 3).

## Discussion

In this study, we comprehensively evaluated the incidence, prevalence, mortality, and DALYs of RHD in China and worldwide over the past 30 years, based on the GBD 2019 database. We compared the differences in disease burden of RHD in China by age and gender. The results showed that the ASIR, ASPR, ASMR, and ASDR of RHD in China all decreased from 1990 to 2019, while the ASIR and ASPR globally increased, and the ASMR and ASDR decreased. The incidence, prevalence, mortality, and DALYs of RHD were related to the age of the patients, with RHD generally prevalent in the younger population, high incidence in the youth, and high mortality in the elderly. In terms of gender composition, females were more susceptible to RHD infection and had a higher risk of death after infection compared to males.

Over the past few decades, with the improvement in living standards worldwide, the widespread access to healthcare, and the extensive use of penicillin-type drugs, the disease burden of RHD has been decreased [4]. The findings of this study indicate a significant decline in ASMR and ASDR globally from 1990 to 2019, which can be attributed to concerted efforts over the past 30 years on RHD disease control, such as enhancing healthcare resources, effective prevention and control measures, and international collaboration [15–17]. Furthermore, low-cost remote healthcare models have also facilitated the screening of RHD patients in low- and middle-income countries, thus reducing ASMR and ASDR [18].

However, globally, the ASIR and ASPR continue to show an upward trend. RHD remains a leading cause of severe valvular heart disease, and untreated RHD can lead to death in patients [19], exacerbating the health burden in certain regions and populations [20]. According to related literature, the incidence and prevalence of RHD are on a significant rise, driven by factors including poverty and limited healthcare resources [21], as well as genetic susceptibility to RHD [22]. Therefore, the global burden of RHD is yet to be adequately controlled, necessitating the continued implementation of targeted, effective, and precise strategies to manage this disease burden.

The burden of RHD exhibits significant regional variations. A study stratifying countries and regions based on the Socio-Demographic Index (SDI) to compare RHD burden across different age groups reported similar findings. The incidence and prevalence of RHD increase in low SDI regions while showing a decreasing trend in middle to high SDI regions. Additionally, the ASDR of RHD significantly decreases in these regions [2]. In this study, the ASDR for RHD in China from 1990 to 2019 decreased by 57.56%, and the ASMR decreased by 47.40%, aligning with reported decreases in ASDR and ASMR in countries with middle SDI. This decline is attributed to the Community-Based Comprehensive Risk Management Project for Cardiovascular Diseases implemented in China in 2015, alongside improvements in lifestyle and public health conditions over the past 30 years. More RHD cases are being detected and treated early [23]. Ge et al. [24] conducted a time-series study exploring the association between environmental temperature and daily RHD admissions in Shanghai, China, finding a positive correlation between daily average temperature and RHD admission rates. Age, gender, and regional (urban, rural) RHD mortality data from the *China Health Statistics Yearbook* indicate higher RHD mortality rates in rural areas compared to urban areas, higher mortality rates in females than males, and a higher risk of RHD-related deaths in the elderly (age 60 and above) [25]. Moreover, significant variations were observed between different countries and regions globally, as documented by the GBD database. Epidemiological studies reported the central region of sub-Saharan Africa, specifically the Sahel region, to have the highest ASIR in the epidemiological study [16]. GBD research from 2020 indicated that India accounted for one-third of the global RHD burden [26]. An epidemiological study focusing on the Americas revealed a 51% lower death rate and a 30% lower prevalence of RHD compared to the corresponding global estimates, with a significant downward trend in mortality from 1990 to 2019 [27]. Thus, the reasons for varying RHD disease burdens within different countries and regions, even within the same country, are likely associated with factors such as socio-economic development levels, geographical environmental factors, genetic and ethnic factors, as well as healthcare resources and vaccination rates [28].

This study found that RHD is more common in people under 50 years old, with the peak incidence occurring between the ages of 0–14 years old, and the peak prevalence occurring between the ages of 20–24 years old and 30–34 years old. Lv et al. [2] also showed that the incidence of RHD is concentrated in the age groups of 5–24 years old and 15–49 years old. Ghamari et al. [19] showed that the incidence of RHD is highest in the 15–19 age group, and timely medical intervention can significantly reduce the mortality rate of RHD. These results indicate that the middle-aged population is susceptible to RHD, highlighting the importance of early screening for RHD. Additionally, health management should be strengthened for people under 50 years old to prevent the development of RHD. The aging population in China and changes in residents’ lifestyles have made chronic non-communicable diseases such as cardiovascular diseases a threat to people’s health and life [29]. Here, the DALYs and mortality rate of RHD were higher in the elderly population and showed an increasing trend with age, with the highest mortality rate occurring in the 95 + age group. This result was similar to that reported by Julie et al. [30] in high-income regions, where the DALYs of RHD increase with age and reach a peak at ≥ 85 years old. This may be linked to the decreased immune function and weakened resistance of the elderly population, leading to increased susceptibility to autoimmune diseases.

In this study, it was observed that females were more susceptible to RHD infection compared with males, and they also face a higher risk of mortality following infection. The reasons behind this phenomenon remain unclear. Reports suggest that this gender disparity may be attributed to factors such as females’ inherent immunological susceptibility, hemodynamic changes during pregnancy, and increased likelihood of contracting Group A streptococcus due to their close involvement in childcare [31]. Additionally, research has indicated that women of childbearing age are more prone to RHD [32, 33]. In low and middle SDI regions, the prevalence of RHD among women of childbearing age is higher compared with affluent areas [34, 35]. This phenomenon is likely due to factors such as limited awareness and education about RHD in girls from impoverished areas, lack of preventive measures, socioeconomic constraints, and limited access to healthcare in remote regions. All these issues warrant careful consideration and highlight the need to strengthen RHD screening for young women and enhance their awareness of RHD as part of disease prevention efforts. Additionally, there should be increased attention to the specific burdens faced by women of childbearing age in impoverished areas, with a focus on conducting cardiac examinations and treatment for high-risk women before and during pregnancy [2].

We conducted a comprehensive analysis of the current status of RHD burden in China and worldwide, focusing on the aspects of incidence, prevalence, mortality, and DALYs between 1990 and 2019. Additionally, the study examined the variations in age and gender burden of RHD in China. The findings of this study offer valuable data for health authorities worldwide, potentially contributing to the global reduction of RHD burden. However, this study has several limitations that need to be acknowledged. Firstly, GBD estimates rely on the quality and quantity of data, such as disease diagnoses and measurements of environmental risk factors over time. In regions with limited access to healthcare and among economically disadvantaged populations, disease diagnoses may be insufficient, especially in low and middle SDI areas where RHD screening may be inadequate. Consequently, the data on RHD burden may be underestimated [2]. Moreover, potential biases stemming from misclassification and miscoding of diseases could affect the accuracy and robustness of the results. Secondly, the diagnosis and detection methods for RHD may have varied over time, and differences in data collection and tools across different periods could introduce potential biases into the data. Finally, in this study, global RHD data are presented at a global level to describe the overall burden of RHD. However, the disease burden levels in different countries or regions may vary due to specific factors such as socio-economic development, geographical environmental factors, genetics, ethnicity, and the level of medical resources and vaccine coverage. Therefore, these global data may not be directly applicable as specific reference levels for disease burden in particular countries or regions, and further in-depth analysis is needed based on data specific to each country or region.

### Electronic supplementary material

Below is the link to the electronic supplementary material.

Supplementary Material 1

## Data Availability

The datasets generated and analyzed during the current study are not publicly available, but are available from the corresponding author on reasonable request.

## Declarations

### Methods statement

All methods were carried out in accordance with relevant guidelines and regulations.
