# Supplementary material for: Analysis and comparison of the trends in burden of rheumatic heart disease in China and worldwide from 1990 to 2019
Source: BMC Cardiovasc Disord. 2023 Oct 24;23:517. doi: 10.1186/s12872-023-03552-w (PMC10594932; doi:10.1186/s12872-023-03552-w)

**Supplementary Figure 1 Comparative chart of global incidence, prevalence, deaths and age-standardized rates of DALYs in various age groups between 1990 and 2019**

(a-d) Comparison of incidence, prevalence, mortality and DALYs by age group.


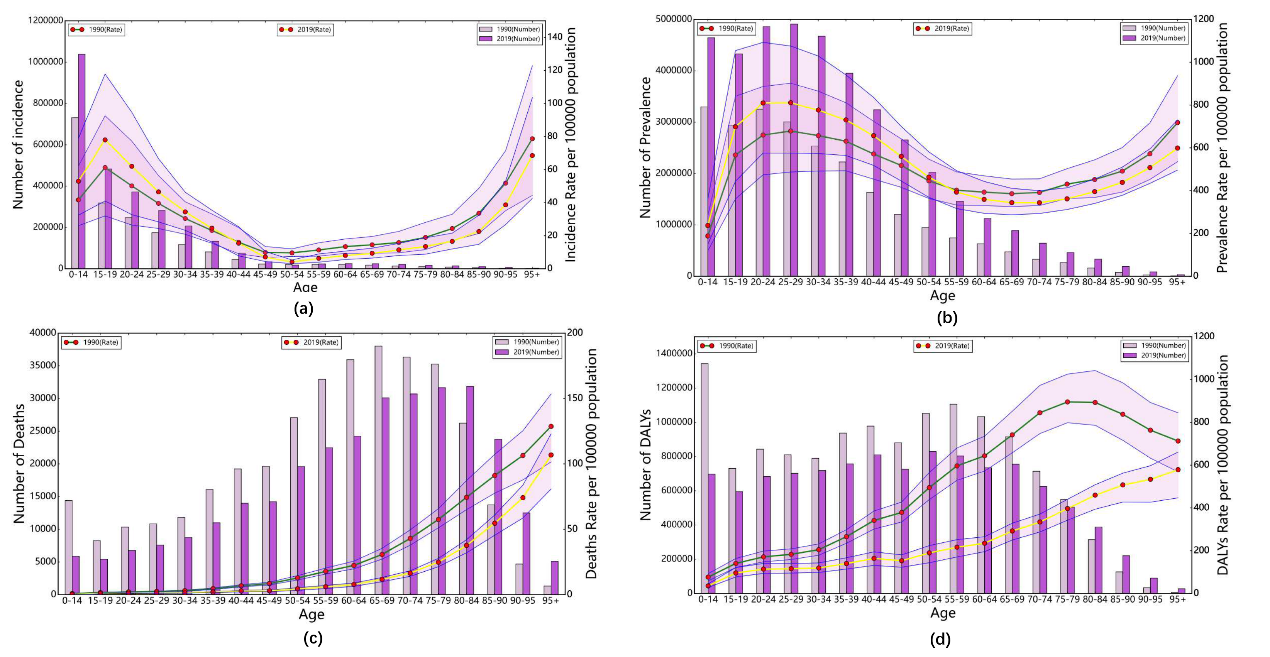


**Supplementary Figure 2 Comparison of incidence, illness, death and DALYs among men and women in various age groups around the world in 1990 and 2019**

(a-d) Comparison of the number of incidences, illnesses, deaths and DALYs between men and women in 1990;

(e-h) Comparison of the number of incidences, illnesses, deaths and DALYs between men and women in 2019.


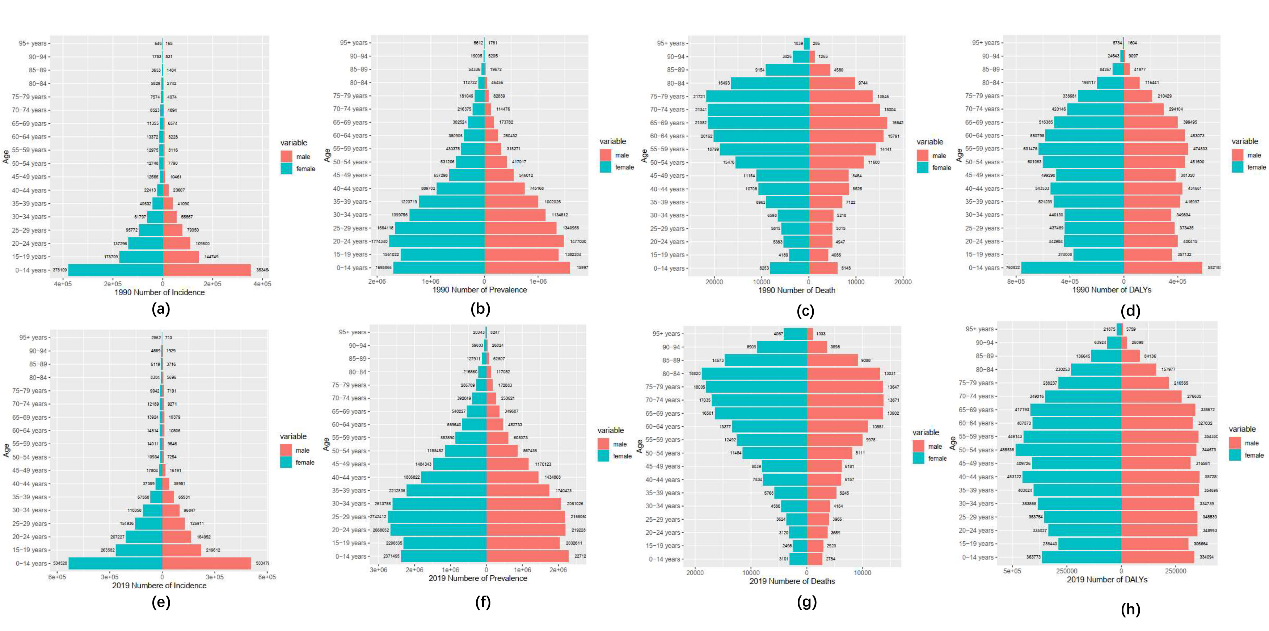


**Supplementary 3 Comparison of all-age cases and age-standardized rates of incidence, morbidity, death and DALYs for men and women globally from 1990 to 2019**

(a-d) Age-wide numbers and age-standardized rates of incidence, prevalence, death and DALYs from 1990 to 2019.


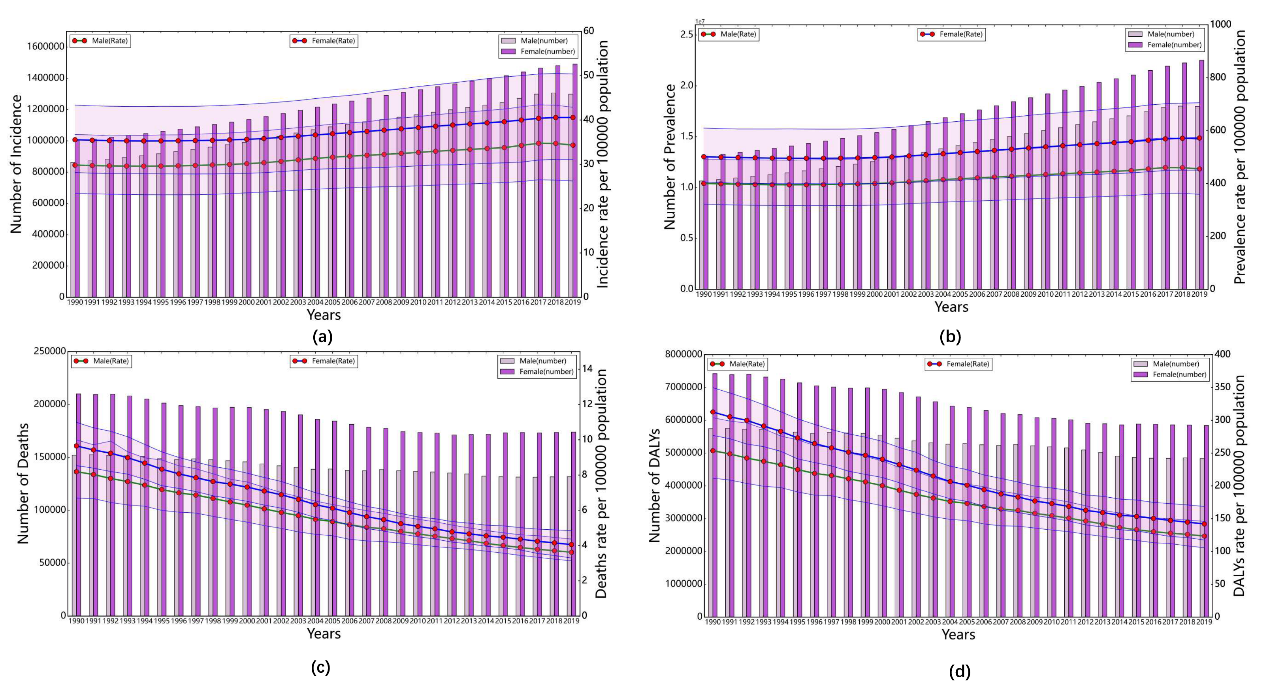

Supplement: Supplementary file 1 — Supplementary Material 1 [file 12872_2023_3552_MOESM1_ESM.docx]
